# Formulation of New Algorithmics for miRNAs

**DOI:** 10.2174/1874357900802010037

**Published:** 2008-04-25

**Authors:** Yoichi Robertus Fujii

**Affiliations:** Nagoya City University, Nagoya, 467-8603, Japan

**Keywords:** AIDS, HIV, microRNA, nef, non-coding RNA, retrotransposon, RNA interference.

## Abstract

microRNAs (miRNAs) are a class of small RNAs, 21-25 nucleotides (nts) long with single-stranded RNA. miRNA targets the sequences of messenger RNA (mRNA) through incomplete base-pairing of the target sequence. The incomplete pairing of miRNA to mRNA triggers either translational repression or epigenetically mediated transcriptional gene silencing (TGS). miRNA and RNA silencing in mammalian cells may participate in natural ecological interactions and miRNA itself should contain the original information that is required to control viral proliferation, according to the hypothesis of RNA waves. While the hypothesis involves so-called resident and genomic miRNA as the genetic information, resident miRNAs may evolve and jump into other RNAs, and then become genomic miRNAs. Thus, the inheritable character may be acquired by both types of miRNAs. It is reasonable to believe that preparations of new algorithmics models for the flow of miRNAs may provide an opportunity to overcome the acquired immunodeficiency syndrome (AIDS) pandemic.

## INTRODUCTION

Elucidation of the mechanisms by which pathogenicity develops is a major issue in virology today. DNA and RNA viruses, excluding viroids, transcribe genetic information to viral messenger RNA (mRNA), and the translation of mRNAs following the production of virally functional proteins is known to cause viral pathogenicity in host cells. The same processes and tools are used in viral protein synthesis in host cells as in the host protein factory, therefore, it is believed that virally functional proteins can render cytopathic effects to virally infected cells. A pathogenic viral particle such as human immunodeficiency virus type 1 (HIV-1) is constructed of viral proteins in cells that assemble, bud, and then transmit the progeny to other cells. Identified in 1983, HIV-1 causes acquired immunodeficiency syndrome (AIDS). In infected T lymphocytes, HIV-1 produces the structural proteins Gag, Pol and Env, which are crucial to establish a full infection. Further, Tat and Rev regulatory proteins have key roles in the control of host factor-dependent HIV-1 replication [1]. However, HIV-1 genomic RNA is only responsible for information concerning the encoding of the viral protein. To date, no studies have focused on the function of HIV-1 RNA and the role it plays in producing a full infection.

In 1953, mobile genetic elements (MGEs) has been discovered by a maize researcher [2]. Genetic evolution involves the mutation of chromosomes with mobile genes *via* a two-unit dissociation-activator (*Ds*-*Ac*) system and the alteration of a certain code inheritable during development. The evolutionary development of viral RNA and that of MGE might have been interdependent, but in some cases, tumor cells have been linked to the transposition or insertion of cellular oncogenes (c-*onc*) as MGE, as well as of viral oncogenes (v-*onc*) as RNA retroviruses. Thus, we must consider the possibility that viral RNA might change the phenotypes of cells by delivering MGEs. In this scenario, retrotransposons including MGEs from the human genome might work with microRNA (miRNA).

miRNA is an approximately 22-nucleotide (nt) small RNA transcribed from a stem-loop structure as non-coding RNAs (ncRNAs), and they can originate from intergenic, introgenic and protein-coding regions of the human genome as well as from viral genomes [3]. miRNA suppresses gene expression at translation and transcription stages without destroying target mRNAs. Since miRNAs are paired to mRNAs with imperfect base-pairing, miRNA can target multiple mRNAs and at the same time each target can be controlled by multiple miRNAs. When the ‘seed’ sequences (7 nts) at the 5’ end of miRNAs are completely matched with its target sequences, the targeted mRNA is cleaved through RNA interference (RNAi) machinery in a process similar that involving short interfering RNA (siRNA). Consequently, 30-50% of host genes might be controlled by miRNAs [4]. Although a prototype of miRNA for *C. elegans* was described in the course of an investigation of nematode development, this was before the discovery of RNAi in 1998 [5-7]. However, viral miRNA has also been found independently in HIV-1 infected human cells in a study aiming to clarify the mechanisms of promoter interference in latent infections. In a study undertaken to elucidate the phenomenon of promoter interference in HIV-1 infection, it was shown that small RNAs from the HIV-1 *nef* /3’-long terminal repeat (LTR) of AIDS patients inhibit both the transcription of HIV-1 and its ability to survive in cells. In addition, genomic miRNAs in host cells suppress the translation of HIV-1 mRNA [1, 4]. The latter discovery is not widely known, but it suggests that miRNA plays a key role in enabling the mobility of genetic information in mammals. Approximately 45% of the human genome, for example, is comprised of retroelements including SINE, LINE, transposons and retrotransposons that are also involved with HIV-1 proviruses in infected individuals [8]. Furthermore, in the social amoeba, DIRS-1 and Skipper retrotransposons encode miRNAs as well as repeat-associated short interfering RNAs (rasiRNAs or piRNAs) during cell formation [9].

**Fig. (1):**
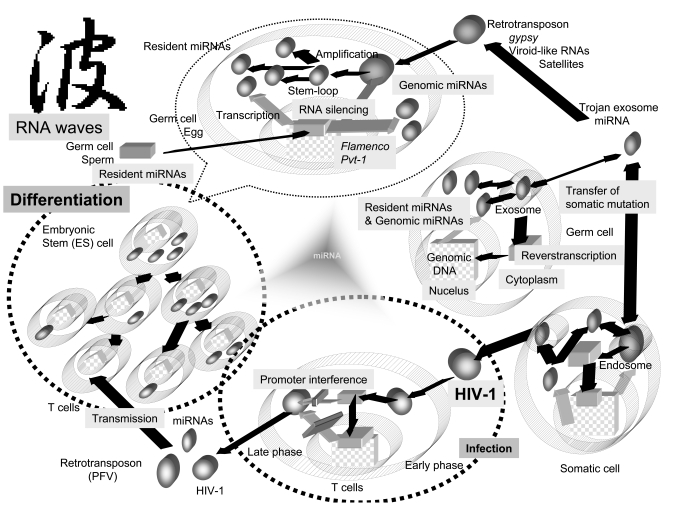
Diagram of mobile miRNAs under the RNA wave hypothesis. Two categories of miRNAs, resident and genomic miRNAs are represented. RNA and DNA information is shown in circles and squares, respectively. Genomic DNA of human cells is represented by large checkered squares. Black arrows represent the flow of RNA and gray arrows that of RNA intermediates from transcription of DNA information. When retrotransposons, viroid-like RNAs and satellites horizontally infect germ cells, the RNA intermediates are a source of small RNA through RNA silencing. The resident miRNAs derived from small RNA are reverse -transcribed and become genomic miRNAs. The genomic miRNAs are vertically inherited and the resident miRNA information is pooled into the host genome. Further, miRNAs are horizontally transmitted by Trojan exosomes between germ and somatic cells, or among these cells. Some miRNAs, such as HIV-1 miRNAs, jump into MGE tags, and miRNAs in MGE can infected blood cells. The information of miRNA in MGE on the genome is silenced by promoter interference. On the other hand, since the represented genetic information would be involved in the resident miRNA, divided ES cells would have asymmetric and inheritable characters if the resident miRNA information is asymmetric. Therefore, the combination of infection of viruses and MGEs with miRNAs may contribute to the elucidation of evolution and differentiation of cells. For example, these aspects may partially explain the imprinting phenomenon and the susceptibility of T cells and macrophages to HIV-1. The Japanese Kanji character ‘nami’ in the top left of the figure is the symbol for waves.

The role of miRNA in gene control is implicated in the processes of cellular development, differentiation, aging, oncogenensis (including stem cell maintenance), and house-keeping metabolisms [4]. All of these processes require the delivery of precise information in which the amplification of regulatory miRNAs may play an important role. Although RNA-directed RNA polymerase (RdRP) has been shown to amplify the effect of RNA silencing in nematodes and plants, it has not been identified in mammalian cells [4]. In mammalian cells, intriguingly, cellular DNA-dependent RNA polymerase II has been shown to carry out the function of RdRP in hepatitis delta virus (HDV) in mammalian cells [4]. However, RdRP activity of endogenous reverse-transcriptase (RT) has also been demonstrated *in vitro* [4], therefore it is also possible that small RNAs including miRNAs and piRNAs can be self-replicated in mammalian cells. Thus, our hypothesis consists of four concepts: 1) infection induces miRNA production in the virus and/or the host; 2) the induced miRNAs have MGE-like mobility; 3) the mobile miRNAs can self-proliferate; and 4) cells contain both resident and genomic miRNAs. Based on those four concepts, we have established a new RNA waves theory that asserts not only can miRNA mediate RNA silencing to control expression MGE but also that miRNA may be necessary to inclusively incorporate MGE for evolution of the genome. The function of viral and mammalian miRNAs still remains unclear. Their involvement in both infection and MGEs implies that their role is a complex one; however, if we could link these two functions using new algorithmics, we could demonstrate that miRNA information flow proceeds through asymmetric division and provides evolutionary advantages in the niches that individuals have filled (Fig. **1**).

The rest of this paper is organized as follows. In the first section, we introduce the origin of mobile small RNAs and discuss the coincidence of resident and genomic miRNAs. In the second, we describe the characters of resident miRNA that are related to HIV-1 latency. Finally, we introduce new algorithmic models for viruses using miRNA information flow, which we hope will contribute to viral therapy and/or prevention. I intend to argue for this ‘RNA wave’ hypothesis and introduce it as a general prospective in our Open Virology Journal.

## TRANSMISSION OF PATHOGENIC AND FUNCTIONAL MIRNAS FROM VIRUSES TO HOSTS

The origin of functional miRNAs vulnerable to pathogenic RNAs may be determined from the study of viroids. Viroids are plant pathogens possessing only single-stranded RNA genomes. The genome consists of 200-400 nt and is an ncRNA [10]. Viroid RNA can be used as the predominant *in vivo* substrate for cleavage by Dicer, and viroid-derived small RNAs have been detected during infection [10]. This evidence suggests that viral small RNAs may contribute to viroid pathogenicity by silencing RNA and thereby silencing plant genes. It is widely accepted that viroid small RNAs in plant cells completely destroy their target mRNAs through RNA silencing [10]. Therefore, completely paired viroid small RNAs can be regarded as siRNAs, although the characters of these siRNAs closely resemble the characters of miRNA because the viroid RNA is not completely cleaved by the miRNA. Further, viroid-like elements known as retroviroids have been found in the genomic DNA of plants. Retroviroids probably make use of RT to integrate some of their own elements into the genome [11]. Thus, viroid small RNAs are the resident miRNA-like intermediates formed in the cytoplasm of cells.

Several small RNAs emerging from ncRNAs have been reported in eukaryotes [4]: (i) siRNAs produced by centromeric repeats in* Schizosaccharomyces pombe*; (ii) scanRNAs (scnRNAs) in *Tetrahymena thermophila*; (iii) siRNAs from transposons in protozoa; (iv) siRNAs produced by *Caenorhabditis elegans*; (v) rasiRNAs from *Drosophila melanogaster*; and (vi) miRNAs in mammalian cells. rasiRNAs repress replication of the *gypsy* virus, an endogenous retrovirus of *D. melanogaster*. Host miRNAs, as genomic miRNA, suppress HIV-1 replication [12], therefore, this type of repression in retroelements is thought to involve in a RNA-silencing mechanism that is dependent on host genomic alleles, such as *flamenco* [13]. Since rasiRNA sequences are complementary to those of the 5’-untranslated region (UTR) of the *gypsy* retrovirus [14], rasiRNAs might be viral miRNA-like intermediates. However, the level of *gypsy* infection is determined by the *flamenco* locus of follicle germ cells, so it is likely that *flamenco* is a heterochromatic RNA-silencing gene for *gypsy* infection involved in the production of rasiRNAs in the ovary. Further, rasiRNAs have also been identified from a variety of repetitive elements including LTRs and non-LTR retrotransposons, transposons, satellites and microsatellites of *D. melanogaster* and *Trypanosoma brucei*, and mouse and human cells [15]. Thus, rasiRNA may be a type of small RNA that is both resident and genomic. The functional characters of miRNAs, siRNAs and rasiRNAs are likely similar, and specifically, they are all likely borderless. A similar process may be involved in mammalian cell (Fig. **1**).

The *Pvt1* locus (known *PVT1* as in humans) is a common retroviral integration site in induced T lymphomas in rodents [16]. Murine leukemia virus (MLV) can integrate into the *Pvt1,* and the provirus acts as integration site “tags” with its own sequences. By proviral tagging, c-*onc* has been discovered through its homology to v-*onc*. These insertion sites include ncRNAs, such as the oncogenic miRNA, mir-155/*BIC* [17]. mir-155 controls oncogenesis, so it is possible that miRNAs encoded by *Pvt1* are also oncogenic. On the other hand, viral miRNAs are expressed not only by oncogenic RNA viruses but also by tumorigenic DNA viruses. Clusters of miRNAs have been identified from herpesviruses such as Epstein-Barr virus (EBV), the Kaposi sarcoma-associated virus (KSHV), the mouse gammaherpesvirus (MHV) and the human cytomegalovirus (HCMV) [18]. For example, 17 miRNAs have been cloned from KSHV-infected primary effusion lymphoma cells. The presence of KSHV miRNAs alters gene expression in the host B cells. Of these viral miRNAs, mir-K12-11 KSHV miRNA has quite recently been reported to show significant homology to oncogenic mir-155/*BIC* [19]. As KSHV is never integrated into its host’s genome, KSHV miRNAs are resident miRNAs. β–herpesvirus HCMV encodes viral miRNAs; one of these, mir-UL112, can suppress the expression of multiple viral genes through target sites within the 3’-UTR of the viral transcript rather than through RNA degradation [20]. These results also suggest that since HCMV is not integrated into its host’s genome, the HCMV DNA genome corresponds to episomal HIV-1 DNA, which may be inhibited by promoter interference, and therefore, HCMV miRNAs are resident miRNAs. It is assumed that resident miRNAs could be reverse-transcribed and then integrated into its host’s genome, as are retroviroids. In that case, the integrated miRNA would act as genomic miRNA with tags that induce oncogenesis. Individuals infected with the above-mentioned herpesviruses experience periods of virtually latent infection [21]. Therefore, virally encoded miRNAs might have contributed to pathogenesis and tumorigenesis in these infected hosts. A similar process might occur in HIV-1 infection.

## CODE AND CIPHER OF miRNAs

A common feature of HIV-1 infection is latency [1]. Latency in infected patients will never be fully understood without models of latency in cell culture techniques. Cell lines that harbor the provirus have been cultured, but they produce only small numbers of viral progeny as persistently infected lymphocytes and macrophages [1]. The HIV LTRs are deeply involved in the modulation of viral gene expression through promoter and enhancer elements contained in the LTR. It is generally known that the 5’- and 3’-LTR activities are regulated by the intense crosstalk of each LTR. Remarkably, the 5’-LTR is not only downregulated by promoter interference from the 3’-LTR but it might also be suppressed in the presence of the 3’-LTR (Fig. **1**). In the case of HIV-1, even in the presence of Tat, the 5’-LTR activity is reduced within a few hours after induction with Tat. These mechanisms may contribute to the latency; however, the mechanisms of the downregulation caused by Tat activation are not clear. Of course, the viral latency is not regulated solely by the LTR activity; however, HIV-1 is completely productive without the promoter interference in Dicer- and Drosha-restricted peripheral blood mononuclear cells (PBMC) [22]. The Tat responsive region (TAR) loop structure has been shown to be a substrate of Dicer, and miRNAs are generated from the TAR RNA. Furthermore, the negative factor (*nef*) gene of HIV-1 is located at the 3’ terminal of the HIV-1 genome and overlaps into the 3’-LTR [23]. The *nef* is so named because it suppresses viral transcription from the LTR. The *nef*/U3 region in the LTR encodes a miRNA, *mir-N367*, and the *nef* long-hairpin RNA inhibits laboratory-established and wild-type HIV-1 production [24]. These results suggest that the miRNA-mediated RNA silencing pathway is involved in HIV-1 latency [25].

HIV-1 is integrated into the host genome; however, many studies have suggested that unintegrated HIV-1 DNA is transcribed to viral RNAs. Interestingly, within 3 hours of HIV-1 post infection, a small amount of multiply cleaved viral RNA was detected in infected cells [26]. The level of the viral RNA detected was not changed by the presence of AZT, an RT inhibitor. These data suggest that virally resident pre-miRNA may remain in the infected cell cytoplasm after infection, and that RNA silencing machinery may use viral miRNA to inhibit production of progeny from episomal provirus DNA (Fig. **1**). Genetic studies on the viroid have revealed that MGEs are depressed by RNA silencing, just as they are by promoter interference. The Tc1 transposon in the genome of *C. elegans* was suppressed in an RNAi-dependent manner, and retrotransposon silencing in *S. pombe* was dependent on an intact RNAi pathway. Further, the murine endogenous retrovirus-L (MuERV-L) and intracisternal A particle (IAP) retrotransposons were silenced by Dicer expression in embryos. Abundant L1 transcripts were obtained from Dicer knockout (KO) mouse embryonic stem (ES) cells [15]. On the other hand, retroviruses induce tumors, activating the expression of miRNAs and telomerase RT. In a previous study, telomerase RT activity was upregulated but telomere length did not change [27]. As RT has been reported to take on the role of RdRP activity *in vitro*, telomerase and endogenous retrovirus RTs may also amplify the resident miRNAs, and the amplified aberrant miRNAs might also positively augment unexpected host oncogene expression while still under the promoter interference. Further, not only the guide sequences including the seed of the miRNA, but also the passenger sequences of a pre-miRNA are available for the functional miRNAs [28], and both miRNAs can target each other in an attempt to control one another [29]. These results indicate that resident miRNAs have the following characteristics: 1) resident miRNAs behave as through they are inherently aware of their function; 2) miRNAs interact with each other *in situ*; and 3) miRNAs have nucleotide sequences of intermediate length.

## QUANTUM THEORY OF RNA WAVES

**Fig. (3):**
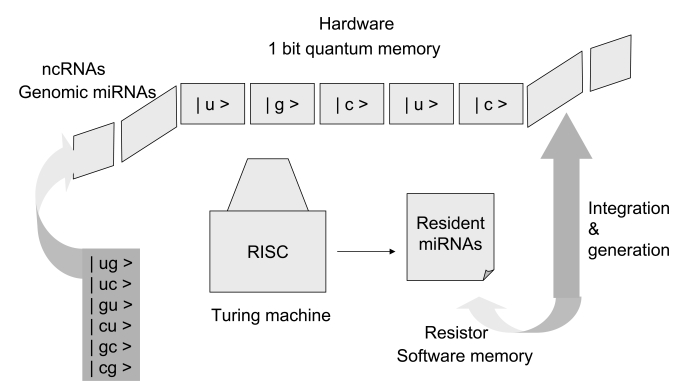
Quantum computation steps with miRNAs. Basically, two resources, computation steps (time) and memory (space), limit the ability of digital computers to solve large problems. Computer devices consist of three core components, namely, memory, a resister, and a Turing machine, which was first described by A. Turing in 1936 [38]. Older computer systems can search objects one at a time; however, the quantum computer by Shor [31] can perform polynomial computation with a random number generator. The system of miRNAs may be closely analogous to that of a quantum computer. Seven sequences in the seed of miRNA may simultaneously target multiple miRNAs with incomplete pairing, and therefore, the seed sequences of the genomic miRNA, which are integrated into the host genome corresponding to the new algorithm shown in Fig. 2, may correspond to quantum memory (space). The resident miRNA sequences may be analogous to a resistor. A Turing machine can read and decipher the genomic miRNA code, and then the information from the genomic miRNAs could be stored as a resistor in resident miRNA sequences. The stored information could be uploaded and/or downloaded into the quantum memory from the resistor. Therefore, the miRISC, which reads seed sequences, may correspond to a Turing machine. In the case of quantum computing (time) based on the seed sequences of miRNA, if the seed sequences are composed of the quantum bits | 0 > and | 1 > ( | u >, | g >, | c > or | ug >, | uc >, | cg > etc as shown in the panel), seven qubits of memory superpose approximately 27 states. With only 500 human miRNAs as 500 records of quantum memory, about 64,000 targets could be selected at one time. Since there are fewer than 20,000 human protein coding genes, the genes can effectively be simultaneously controlled by miRNAs. The complete decipherment of the miRNA code has only now begun.

The phenotypic expression of individual cells by miRNA modulation may be applicable to cellular automata through compatible mathematical models. As shown in Fig. (**2**), the genetic flow of resident and genomic miRNAs can be given simply. In the field of RNA, it is well known that the original code is derived from the two bases guanine (g) and cytosine (c). As the seed sequences in miRNAs do not need to be conserved into the sequences of other miRNAs, the two bases plus uridine (u) and inosine (i) may be randomly aligned into the original seed. Then, the original seed could make the linkage with each other. Resident miRNAs may be evolved and jumped into other RNAs and then turn to genomic miRNAs. In the proposed algorithm, there are two gates, RNA silencing and promoter interference gates, necessary for selection of passes with AND (YES) or NOT (NO) transformation. The RNA silencing pathway is the incomplete process. Therefore, the promoter interference gate can back up the RNA silencing gate and the promoter interference gate is reversible. When miRNAs pass through the RNA silencing gate, the miRNA information contained in MGEs is recorded into resident miRNAs. When miRNAs do not pass through the RNA silencing gate, the miRNAs in the MGEs are integrated into the host genome. When the integrated miRNAs pass through the promoter interference gate, the miRNA information is recorded into genomic miRNAs. When integrated miRNAs do not pass through the promoter interference gate, the miRNAs in retroelement tags can be back to the orphan MGE. During these processes, miRNAs are inherited as asymmetric memory (software) or symmetric memory (hardware). Resident miRNAs are inherited as the former and genomic miRNAs as the latter. The miRNA-induced silencing complex (miRISC) deciphers and binds information from both resident and genomic miRNAs. Although the direction and energy of the sequences borne on miRNA can be calculated in linear models, the mechanism of miRNA information flow might correspond to nonlinear mapping models based on deterministic chaos [30]. Therefore, the phenotypic expression may be applicable to cellular automata. If two nucleotides corresponding to | 0 > or | 1 > bit are aligned into the seed sequences and are represented in the superposition of states, the memory system of miRNAs may be analogous to that of a quantum computer (Fig. **3**) [31].

Regarding these memories, the resident miRNAs may be a communicative tool among the cells, since recent studies have shown that secretory exosomes, such as Trojan exosomes and microvesicles shed by cells contain a subset of the resident miRNAs and RNA transcripts [32], which can be transferred to recipient cells under the RNA waves (Fig. **1**). Since multiply spliced HIV-1 RNAs have been found in the nuclei of resting CD4+ T cells from patients undergoing highly active antiretroviral therapy (HAART) [33] and the sperm nuclei have been reported to contain miRNAs [34], these results support the assertion that resident miRNAs are present and can change to MGEs, and then the resident miRNAs may become acquired inheritable characters. Further, some reports have argued that stem cells express individual miRNAs differentially among cell types and that the miRNAs profiles are important for the maintenance and function of each stem cell type [4]. Since mouse embryonic stem (ES) cells in the absence of Dicer display a failure to differentiate, the miRNA pathway must be required for the regulation of stem cell fate [35]. Based on these results, it seems that the resident miRNA lineages may be altered during differentiation (Fig. **1**). These results also suggests that resident miRNA is somatically inherited as ‘software’, and HIV-1 susceptible T lymphocytes and macrophages might lack some miRNA information during hematopoietic cell differentiation as compared with the miRNAs of human germ cells. This loss in some resident miRNAs could cause partial promoter interference. Therefore, HIV-1 retrotransposons can overcome promoter interference and be reactivated. Assuming that complete RNA silencing and promoter interference in germ cells defends against endogenous retroviruses, it appears that the lack of some resident miRNAs in T cells is compensated for, as compared with the germ cell miRNAs. Of course, hematopoietic stem cell transfusion including RNA silencing agents could conceivably be an *ex vivo* method to compensate for the lost miRNAs on the cellular level [36]. Further, the course of HIV-1 infection of T cells and mononuclear phagocytes (MPs) is vastly different. Although infected T cells are killed, MPs are hijacked by HIV-1, which does not kill them. Differences in resident and genomic miRNA expression may reflect the cytopathic effects on each cell lineage.

HIV-1 vaccine trials have produced disappointing results and have now come to a deadlock [37]. More vigorous efforts are urgently needed and options must be offered in HIV-1 vaccine trials. The trials performed were not without benefit; however, the good results of the GEMINI siRNA trial phase II against the respiratory syncytial virus (RSV) have quite recently been cited by Alnylam Pharmaceuticals Inc., including that RNA silencing therapy could be a new thrapy for infectious disease, such as H5N1 influenza RNA virus infection. Further, VRX496 from VIRxSYS Corp., which is a lentivector agent and expresses the RNA antisense against the HIV-1 *env* gene, reduced production of replicative virions through phase II trial of gene therapy for the treatment of AIDS. The characters of antisense RNA is involved in that of the anti-miRNA oligonucleotides (AMOs). A single injection of this AMO had a measurable effect on the HIV-1 replicative fitness for up to 3 years; therefore, the agent does not require daily administration. Thus, miRNA and RNA silencing agents may be effective for AIDS intervention and have the potential to change HIV/AIDS care. The algorithmics of miRNA flow might be useful for intervention and prevention programs. Surely, this now gives us hope that we may overcome the AIDS pandemic despite the failures of AIDS vaccine trials.

## CONCLUSION

We have established a new RNA waves theory that asserts not only can miRNA mediate RNA silencing to control expression MGE but also that miRNA may be necessary to inclusively incorporate MGE for evolution of the genome under promoter interference. This hypothesis consists of four concepts: 1) infection induces miRNA production in the virus and/or the host; 2) the induced miRNAs have MGE-like mobility; 3) the mobile miRNAs can self-proliferate; and 4) cells contain both resident and genomic miRNAs. The mechanisms of miRNA-mediated gene silencing are still under debate; however, the application of mathematical models of miRNA information flow remains. Based on those four concepts, we here introduce a new algorithmic model for retroviral infection with miRNAs. The ability of digital computers was limited by time (computation steps) and space (memory). Similarly, miRNA as genetic information is limited by the memory system (space) of the viral and host genome involving the resident and genomic miRNAs. Further, miRNA memory flow is limited by computation steps (time) *via* RNA silencing and promoter interference gates. Thus, the actual computation steps with miRNAs are analogous to a quantum computation model. The function of miRNAs is represented by the superposition of states as follows.

$\sum_{\text{i}=0}^{4^{\text{n}}-1}{\text{a}}_{\text{i}}|{\text{S}}_{\text{i}}>$

a_i_ represents the amplitude and | S_i_> the basis state. 4^n^ states as basis vectors are corresponding to bases of nucleic acids, U, C, G and I. Further formulation of new algorithmics may be necessary to completely decipher the miRNA code.

## Figures and Tables

**Fig. (2):**
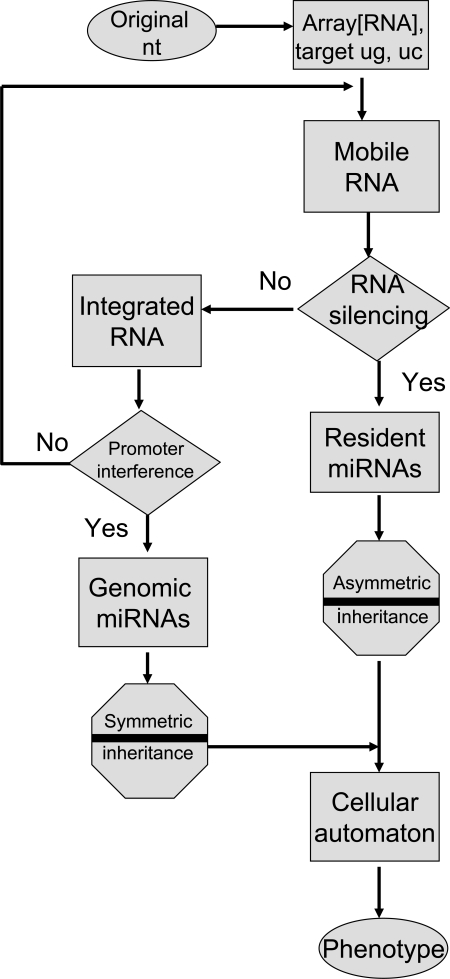
Mobile miRNA memory flowchart. The diagram in Fig. (**1**) is simplified and the resource of space (memory) for computing is shown by the algorithm. In the world of RNA, it is a well-known hypothesis that the original archaic code consists of guanine (g), cytosine (c) and uridine (u). The seed memory of miRNAs in MGE may pass through two gates after infection. One is the RNA silencing gate and the other is the promoter interference gate. When RNA memory passes through the RNA silencing gate (YES), miRNA transforms into resident miRNA as software memory. In the case of NO transformation, miRNAs in the MGEs are integrated into the host genome. When RNA memory in the genome passes through the promoter interference gate, miRNA transforms into genomic miRNA as hardware memory. The genomic miRNAs express a symmetric phenotype and the resident miRNAs express an asymmetric phenotype. Chimerical information of both miRNAs expresses individual phenotype of cells under cellular automaton. miRNA information flow may contribute to the elucidation of differentiation and evolution of cells, such as those involved in mental memory and behavioral diseases.
